# Supplementary material for: Limb myology and muscle architecture of the Indian rhinoceros Rhinoceros unicornis and the white rhinoceros Ceratotherium simum (Mammalia: Rhinocerotidae)
Source: PeerJ. 2021 May 11;9:e11314. doi: 10.7717/peerj.11314 (PMC8121076; doi:10.7717/peerj.11314)
Supplement: Supplemental Information 2 [file peerj-09-11314-s002.docx]

**Additional quantitative comparisons with Haughton (1867)**

The absence of measurements of fascicle length and pennation angle in early works on muscular anatomy, including Haughton’s study of *R. unicornis*, prevents us from doing a true comparison of estimated Fmax. However, muscle mass is available in the study, and additional comparisons are reported here.

Haughton’s specimen was three years old, a young age for a rhinoceros (Dinerstein, 2003). This explains that its total limb muscle mass is inferior to both our specimens (32kg for the forelimb, 31kg for the hindlimb, vs. 84kg and 86kg for our *R. unicornis* and 67kg and 66kg for our *C. simum*). To compare the data, each muscle’s mass was divided by the total mass of the muscles of the corresponding limb (Table S2).

Patterns are similar between the three individuals, and particularly between the two *R. unicornis*. Muscle mass is heavily concentrated in the proximal parts in all of them. The distal muscles are lighter, suggesting that the strength that body support muscles of the distal segments need come primarily from a high pennation angle with very short fibers, as in our specimens. In the forelimb, Haughton’s individual distinguishes itself by proportionally heavy *pectorales* and light *serrati ventrales*. This is consistent with its young age, as it is possible the *serrati ventrales* do not yet have to counteract the extreme body weight that those of fully-grown adults do. Similarly, the *latissimus dorsi* is proportionally lighter in this specimen, probably due to the trunk being less heavy.

In the hindlimb, it is remarkable that the two *R. unicornis* specimens present a *gluteus superficialis* twice as heavy as the *medius*, whereas the *medius* is more than three times heavier than the superficialis in our *C. simum*. This is consistent with our hypothesis that the propulsive role of the *gluteus medius* is partially taken over by the *superficialis* in this species. The *gluteobiceps* of Haughton’s rhinoceros is particularly light, comprising only 4.4% of the total muscle mass of the hindlimb, compared to 10.7% in our *R. unicornis* and 26.7% in our *C. simum*. The two *R. unicornis* again seem similar in this regard, but the muscle was only incompletely weighed in our specimen. One could suppose that the *gluteobiceps* has an important role in body support, meaning that it is not yet fully developed in a subadult specimen, and that in an old and weak specimen like our *C. simum*, its function might be more essential than that of muscles specialized in propulsion, leading to it taking a more important part of the total mass of the limb. The *flexor digitorum superficialis* of Haughton’s *R. unicornis* is proportionally of similar weight to our specimen, and is not tendinous like in our *C. simum*.

**References**

Dinerstein E. 2003. *The Return of the Unicorns: The Natural History and Conservation of the Greater One-Horned Rhinoceros*. New York: Columbia University Press.

Haughton S. 1867. On the muscular anatomy of the rhinoceros. *Proceedings of the Royal Irish Academy* 9:515–526.
